# Regulation and function of macrophage colony-stimulating factor (CSF1) in the chicken immune system

**DOI:** 10.1016/j.dci.2019.103586

**Published:** 2020-04

**Authors:** Zhiguang Wu, Rakhi Harne, Cosmin Chintoan-Uta, Tuan-Jun Hu, Robert Wallace, Amanda MacCallum, Mark P. Stevens, Pete Kaiser, Adam Balic, David A. Hume

**Affiliations:** a The Roslin Institute, University of Edinburgh, Easter Bush, Midlothian, EH25 9RG, UK; b The Department of Orthopedic Surgery, University of Edinburgh, Chancellor's Building, Edinburgh BioQuarter, 49 Little France Crescent, Edinburgh, EH16 4SB, UK; c Mater Research Institute-University of Queensland, Translational Research Institute, Woolloongabba, QLD, 4104, Australia

**Keywords:** Macrophages, CSF1, Chicken, Neutralising antibody

## Abstract

Macrophage colony-stimulating factor (CSF1) is an essential growth factor to control the proliferation, differentiation and survival of cells of the macrophage lineage in vertebrates. We have previously produced a recombinant chicken CSF1-Fc fusion protein and administrated it to birds which produced a substantial expansion of tissue macrophage populations. To further study the biology of CSF1 in the chicken, here we generated anti-chicken CSF1 antibodies (ROS-AV181 and 183) using CSF1-Fc as an immunogen. The specific binding of each monoclonal antibody was confirmed by ELISA, Western blotting and immunohistochemistry on tissue sections. Using the anti-CSF1 antibodies, we show that chicken bone marrow derived macrophages (BMDM) express CSF1 on their surface, and that the level appears to be regulated further by exogenous CSF1. By capture ELISA circulating CSF1 levels increased transiently in both layer and broiler embryos around the day of hatch. The levels of CSF1 in broilers was higher than in layers during the first week after hatch. Antibody ROS-AV183 was able to block CSF1 biological activity *in vitro* and treatment of hatchlings using this neutralising antibody *in vivo* impacted on some tissue macrophage populations, but not blood monocytes. After anti-CSF1 treatment, *CSF1R*-transgene reporter expressing cells were reduced in the bursa of Fabricius and cecal tonsil and TIM4^+^ Kupffer cells in the liver were almost completely ablated. Anti-CSF1 treatment also produced a reduction in overall bone density, trabecular volume and TRAP^+^ osteoclasts. Our novel neutralising antibody provides a new tool to study the roles of CSF1 in birds.

## Highlights

• Two mouse monoclonal antibodies to chicken CSF1 (ROS-AV181 and 183) were produced.
• A capture ELISA assays was developed to quantify native circulating CSF1 levels.
• Antibody ROS-AV183 was shown to neutralise the bioactivities of CSF1 *in vitro.*
• Treatment of hatchlings using ROS-AV183 *in vivo* impacted on chicken macrophage subsets and B-cells.
• Anti-CSF1 treatment produced a reduction in overall bone density, trabecular volume and TRAP^+^ osteoclasts.

## 1 Introduction

Macrophage colony-stimulating factor (CSF1) controls the proliferation, differentiation and survival of cells of the macrophage lineage in mammals and birds (Chitu and Stanley, 2017; Garceau et al., 2015; Hume et al., 2019; Jenkins and Hume, 2014; Jenkins et al., 2013). CSF1 acts by binding to a tyrosine kinase receptor (CSF1R), which is encoded by the c-fms proto-oncogene (Sherr et al., 1985). Expression of CSF1R is largely restricted to mononuclear phagocytes (Rojo et al., 2017), but in mammals is detected on trophoblast cells (Arceci et al., 1989; Regenstreif and Rossant, 1989) and in chickens it is detected at low levels on heterophil granulocytes (Balic et al., 2014) and antigen sampling follicular epithelial cells in mucosal lymphoid associated tissues (Balic et al., 2019; Sutton et al., 2018). A second ligand for CSF1R, interleukin 34 (IL-34) has been identified (Lin et al., 2008); however, its expression is more tissue restricted than CSF1 (Wang et al., 2012) and the functions and signalling activities of these CSF1R ligands are not identical (Chihara et al., 2010; Liu et al., 2012; Wei et al., 2010). Both chicken CSF-1 and IL-34 were shown to elicit macrophage growth from chicken BM cells in culture (Garceau et al., 2010). We previously produced a recombinant chicken CSF1-Fc fusion protein (Garceau et al., 2015). As in mice (Gow et al., 2014), CSF1-Fc administration to birds produced a substantial expansion of tissue macrophage populations (Garceau et al., 2015). Mutation of *Csf1* in mice (op/op) produces a substantial reduction of tissue macrophage populations alongside pleiotropic impacts on somatic growth and development and variable impacts in cancer and inflammatory disease models (Chitu and Stanley, 2006).

Some of these impacts are mimicked by neonatal treatment with neutralising anti-CSF1 antibody, when CSF1 levels are highest (Wei et al., 2005) or following prolonged treatment of adults with anti-CSF1R antibody (MacDonald et al., 2010; Sauter et al., 2014). More recently, neutralising monoclonal antibodies against mouse CSF1 and IL34 were developed and shown to have selective impacts on tissue macrophage populations *in vivo* (Lin et al., 2019). To support studies of the function of CSF1/CSF1R signalling in birds, we previously generated *CSF1R* reporter transgenic lines (Balic et al., 2014) and an anti-CSF1R antibody (Garcia-Morales et al., 2014). The antibody provides a marker for blood monocytes and tissue macrophages (CD115), but it did not block binding of CSF1 and signalling by the receptor.

In the current study we describe the production and characterisation of a novel anti-chicken CSF1 monoclonal antibody and show its applications in Western blot, capture ELISA and neutralising assays. We show here that in chicken CSF1 is produced at high levels by mature macrophages themselves and contributes to the development of the immune system in the post hatch period. We discuss the extent to which some of these insights may also be applicable to mammalian macrophage development, homeostasis and function.

## 2 Materials and methods

### 2.1 Animal experiments

All birds were obtained from the National Avian Research Facility at The Roslin Institute, University of Edinburgh. Production of the transgenic chicken lines used in this study have been previously described (Balic et al., 2104). All birds were hatched and housed in premises licensed under a UK Home Office Establishment License in full compliance with the Animals (Scientific Procedures) Act 1986 and the Code of Practice for Housing and Care of Animals Bred, Supplied or Used for Scientific Purposes. All procedures were conducted under Home Office project licences PPL 70/8940 (for transgenic animals; Prof. Helen Sang) and PPL 60/4420 (for administration of substances; Prof. Mark Stevens), according to the requirements of the Animal (Scientific Procedures) Act 1986, with the approval of local ethical review committees. Animals were humanely culled in accordance with Schedule 1 of the Animals (Scientific Procedures) Act 1986.

### 2.2 Monoclonal antibody production, purification, isotyping, and labelling

Chimeric CSF1-Fc fusion protein was produced as previously described (Garceau et al., 2015). The fusion protein has the minimal active fragment of chicken CSF1 and the chicken IgY constant heavy chain domains 3 and 4. ChCSF1-Fc was used as immunogen to immunise mice. Immunisation and fusion to generate hybridomas was carried out by Dundee Cell Products (DCP, Dundee, UK). Following fusion, hybridoma supernatants were screened by indirect ELISA for reactivity with recombinant chCSF1-Fc or diluted chicken serum (as IgY-Fc control). Indirect ELISA was performed as described previously (Rothwell et al., 2001). Briefly, assay plates (Nunc Immuno MaxiSorp, Thermo Fisher Scientific, UK) were coated with recombinant chCSF1 or diluted chicken serum in Carbonate/Bicarbonate buffer at 4 °C overnight. Hybridoma cultures or purified mAb were added after the pre-coated plates were washed and blocked with 0.5% (w/v) casein in phosphate-buffered saline (PBS). After three washes with PBS, plates were then incubated with goat anti-mouse Ig-horseradish peroxidase (HRP; 1/1000, Southern Biotech, Cambridge, UK) at room temperature (RT) for 1 h. Peroxidase activity was developed by adding trimethylbenzedine (TMB) substrate (Thermo Fisher Scientific). The reaction was stopped by 2N sulfuric acid stop solution. Plates were read at 450 nm in a SpectraMax 250 microplate spectrophotometer system (Molecular Devices, Sunnyvale, CA, USA). Positive wells were single-cell cloned. Two monoclonal antibodies, designated as ROS-AV181 and ROS-AV183 were purified using HiTrap Protein G affinity columns and dialysed against PBS using 30 kDa molecular weight cut-off (MWCO) Slide-A-Lyser cassettes (Pierce, Thermo Fisher Scientific). The concentrations of mAbs were determined by absorbance at 280 nm with a Nanodrop. The antibody was isotyped using the IsoStrip Mouse Monoclonal Antibody Isotyping Kit (Roche) according to the manufacturer's instructions. Purified antibodies were biotinylated using Sulfo–NHS–LC-LC-biotin (Thermo Fisher Scientific) and desalted to remove non-reacted biotin using Zeba™ Spin Desalting Columns (Thermo Fisher Scientific) as per instructions.

For *in vivo* studies, large volumes of the neutralising mAb (ROS-AV183) and IgG1 isotype control antibody (mouse anti-ovine CD335 (GR13.1)) were purified from hybridoma culture and dialysed as above. Antibody was filtered through a low protein binding syringe 0.2 μm filters (GE Healthcare Whatman™ Puradisc 25 mm syringe filter) for sterilisation.

### 2.3 Development of a capture ELISA assay and comparison of circulating CSF1 in broilers and layers at different ages

To develop a capture ELISA assay, a chequerboard titration method was used to determine the optimal concentration of coating antibody and detecting antibody. After optimisation, a capture ELISA assay was developed using ROS-AV181 as capture antibody and biotinylated ROS-AV183 as detecting antibody. Briefly, assay plates were coated with capture antibody ROS-AV181 at 2 μg/ml overnight at 4 °C. Plates were washed and blocked as in the indirect ELISA. Plates were incubated with test samples at RT for 1 h. Serially diluted recombinant chicken CSF1 (produced in bacteria, Garceau, 2014) was used to generate a standard curve. Plates were washed and incubated with biotinylated detecting antibody ROS-AV183 at 0.25 μg/ml at RT for 1 h. After three washes, plates were incubated with streptavidin–HRP (1:5,000, Pierce) for a further hour at RT before adding substrate (1-step TMB; Thermo Fisher Scientific) and then 2N sulfuric acid stop solution. Absorbance was read at 450 nm.

For comparative analysis of CSF1 levels using the CSF1 ELISA assay, broiler eggs (Ross 308) and layer eggs (Novogen Brown) were bred in the National Avian Research Facility at The Roslin Institute. Embryos were sacrificed for blood on day 19, 20 and chicks on day 1, day 2, day 3, day 7 and day 14. Sera were collected from clotted blood samples after spinning at 2,000 *g* for 10 min.

ELISA results were calculated using Graphpad Prism 6.07. Standard curve was interpolated with Second order polynomial (quadratic).

### 2.4 Western blotting of tissue lysates and sera

Tissues and sera were collected from 8-week-old Novogen brown birds. Approximately 10 mg of tissue was homogenized in 500 μL of cold radioimmunoprecipitation assay buffer (RIPA Buffer) containing protease and phosphatase inhibitors (Roche) using CK mix homogenising tubes and Precellys 24 (Bertin Instrument). After homogenisation, samples were centrifuged at 10,000 *g* for 20 min at 4 °C to pellet cell debris. Protein concentration of total lysates was determined using Pierce bicinchoninic acid (BCA) protein assay kit (Thermo Fisher Scientific) according to the manufacturer's instructions.

Tissue lysate and serum samples were treated with SDS-PAGE reducing buffer, denatured for 5 min at 100 °C and loaded onto 4–20% pre-cast Mini-PROTEAN TGX Gels (Bio-Rad) and transferred onto 0.45 μm pore size Hydrophobic Immobilon-P Transfer Membrane (Millipore) using a tank transfer method according to the tank's manufacturer instructions (Bio-Rad). Blots were then washed in PBS and blocked in 0.5% skimmed milk/PBS for 1 h at RT. After washing three times, blots were incubated in primary antibodies (ROS-AV181, ROS-AV183 or mouse IgG1 control at 1.0 μg/ml) for 1 h at RT. Blots were then incubated with goat anti-mouse IgG1-horseradish peroxidase (1/1000, Southern Biotech) diluted in 0.5% skimmed milk powder/PBS. Detection was carried out using enhanced chemiluminescence (ECL) (GE Healthcare Life Sciences), according to the manufacturer's instructions.

### 2.5 Immunofluorescence staining and confocal imaging

Spleen and bursa were isolated from commercial layer chickens (Hy-Line, UK), washed with PBS and cryo-embedded in Tissue-Tek Optimal Cutting Temperature (OCT) compound (Sakura Finetechnical). These tissues were sectioned at 10 μm on a Leica CM1900 cryostat, placed onto Superfrost Plus (Menzel-Gläser) glass slides and left to dry. Sections were fixed in ice cold methanol for 20 min and blocked for 1 h in blocking solution (10% normal horse serum, 0.1%Triton X-100 in PBS (MST-PBS)) then incubated with anti-CSF1 antibody (ROS-AV181) for 1 h at RT. After washing with PBS, slides were incubated with goat anti-mouse IgG1 Alexa Fluor 647 (Invitrogen, 1/500) prior to nuclear staining with 4′,6-diamidino-2-phenylindole (DAPI. Invitrogen). Slides were washed in PBS for 30 min on a shaker and mounted using Prolong Gold anti-fade mounting solution (Invitrogen). Confocal images were taken on Zeiss LSM 710 inverted microscope and processed using Zen 2012 (Blue Edition, Carl Zeiss Microscopy) or ImageJ software.

Liver samples from *CSF1R*-eGFP transgenic reporter birds after subcutaneous injections (described below) were collected in ice cold PBS and fixed in 4% paraformaldehyde (PFA) overnight, followed by washing in PBS and storage in 30% sucrose until the tissues sank. Tissues were then cryo-embedded in OCT and sectioned for staining with anti-TIM4 (clone JH9, Hu et al., 2016) overnight at 4 °C. Sections were then incubated with secondary antibodies; Donkey anti-mouse IgG (H + L) (Invitrogen) to detect TIM4 and Rabbit anti-GFP Alexa Fluor 488 (Invitrogen) to detect CSF1R-eGFP. Slides were then washed, mounted and visualised as described above. Images were processed using ImageJ. TIM4^+^ cells were counted in five different images from five different areas of liver sections per animal per treatment.

### 2.6 Flow cytometric analysis

CSF1-Fc was prepared as described previously (Garceau et al., 2015). Anti-CSF1 and CSF1-Fc were conjugated to Alexa Fluor 647 (AF647) using the AF647 Microscale Protein labelling kit (Thermo Fisher Scientific) according to manufacturer's instructions.

To measure surface CSF1 on BMDM, chicken BMDMs were cultured from bone marrow cells from hatchlings in the presence of recombinant chicken CSF-1 as previously described (Garceau et al., 2010). On day 6 of culture, cells were refreshed with culture medium with CSF1 or starved of CSF1 for 18 h. On day 7, cells were stimulated with 0.5 μg/ml of lipopolysaccharide (LPS; *E. coli* serotype 055:B5, Sigma) for 3 h or non-stimulated. Cells were washed with cold FACS buffer (PBS/1.0%BSA/0.05% Sodium azide), incubated in anti-CSF1- Alexa Fluor 647 or CSF1-Fc- Alexa Fluor 647 at 4 °C for 30 min.

For flow cytometric analysis of primary cells, single cell suspensions were isolated from blood and spleen. EDTA-treated blood was diluted to 2 ml and gently overlaid onto the same volume of Lymphoprep with a density 1.077 g/ml (STEMcell Technologies) prior to centrifugation at 400*g* without brake for 30 min. Cells at the interface were carefully collected and washed twice with FACS buffer. Spleens were collected in cold PBS, dispersed through a 100 μm cell strainer and isolated by centrifugation over Lymphoprep as described above for blood cells. Cells were then blocked with FACS buffer for 5 min and incubated on ice with monoclonal antibodies listed in Table 1 at appropriate concentrations as determined by titration assays. Cells were washed twice and re-suspended in FACS buffer and analysed using a BD LRSFortessa (BD Biosciences, UK). SYTOX® Blue Dead Cell Stain (1.0 μM, Invitrogen) was added 5 min prior to analysis to exclude dead cells. Doublets were discriminated based on signal processing (SSC-A/H or FSC-A/H). Data were analysed using FlowJo software (FlowJo, Ashlan, OR. USA).

**Table 1:** List of primary and secondary antibodies used for flow cytometry and immunohistochemistry.

Antibody name/Clone	Antigen	Isotype	Supplier	Working concentration μg/ml
Mouse anti-CD45-RPE/clone LT-40	CD45	IgM	Southern Biotech	0.8
Mouse anti-chicken Monocytes/Macrophages-RPE/clone KUL01	MRC1L-B	IgG1	Southern Biotech	0.4
Mouse anti-Bu-1-RPE/clone AV20	Bu-1a/b	IgG1	Southern Biotech	0.1
Mouse anti-chicken MHC II-RPE/clone 2G11	MHC class II β-chain	IgG1	Southern Biotech	0.1
Mouse anti-CD3-RPE/clone CT-3	CD3	IgG1	Southern Biotech	0.1
Mouse anti-CD41/61/clone 11C3	Integrin CD41/61	IgG1	Bio-Rad	0.8
Mouse anti-TIM4/clone JH9	TIM4	IgG1	In house (Hu et al., 2016, 2019)	0.1
Mouse IgG1 isotype control/GR13.1		IgG1	In house (Hu et al., 2016, 2019)	0.1
Mouse IgM isotype control-RPE		IgM	AlphaDiagnostic	0.8
Mouse IgG1 isotype control-RPE		IgG1	AlphaDiagnostic	0.1

### 2.7 Neutralisation of CSF1 activity

To determine the ability of antibodies to neutralise CSF1 *in vitro*, recombinant CSF1 was pre-incubated with anti-CSF1 mAbs or a mouse IgG1 control at 37 °C for 2 h before addition to bone marrow or Ba/F3 cells expressing the chicken CSF1R (Garceau et al., 2010). Chicken bone marrow cells from embryonic day (ED) 20 Novogen embryos were cultured with CSF1 and re-fed every 2–3 days with the same fresh media and incubated for 7 days. Ba/F3 cells expressing chicken CSF1R (Garceau et al., 2010) were maintained in culture with complete RPMI medium supplemented with 10% heat-inactivated foetal bovine serum (FBS), 2 mM l-glutamine and antibiotics (100 g/ml penicillin, 100 g/ml streptomycin) and 5% conditioned medium from X63 Ag8-653 myeloma cells carrying an expression vector for IL-3 (Karasuyama and Melchers, 1988) at 37 °C, 5% CO_2_ prior to assays for cellular metabolic activity using the tetrazolium dye MTT. Cells were washed twice with PBS to remove any IL-3, and plated in a 96 well plate in triplicate at 4 × 10^4^ cells/well in 100 μl of complete RPMI without X63 supernatant. Cells were treated with serial dilutions of chicken CSF1 and incubated for 72 h at 37 °C, 5% CO2. 10 μl of MTT (Sigma-Aldrich) stock solution (5 mg/ml) was added directly to each well to achieve a final concentration of 0.5 mg/ml and incubated at 37 °C for 3 h before adding 100 μl of solubilisation solution (0.1 M HCl, 10% Triton X-100 and isopropanol) and incubation overnight. Plates were read at 570 nm.

### 2.8 Subcutaneous injection of anti-CSF1 in hatchlings

Seventeen newly hatched *CSF1R*-eGFP transgenic chicks (Balic et al., 2014) were weighed and divided into two groups with matching body weight. One group of chicks was injected subcutaneously in the breast muscle region with neutralising anti–CSF–1 antibody (ROS-AV183) (10 mg/kg, ca. 500 μg per bird) on each of four consecutive days. The control group was treated with isotype control antibody (mouse anti-ovine CD335, clone GR13.1). Body weights of all birds were measured on day 1, 2, 3, 4 and day 7. Blood was collected for flow cytometry analysis into a BD Vacutainer collection tube and analysed by Flow Cytometry as above. Lymphoid tissues including spleen, cecal tonsils and bursa were collected and placed in PBS in a Petri dish for whole mount imaging performed using a Zeiss Axio Cam HR fluorescent microscope. Surface area of the lymphoid tissues and mean fluorescence intensities of *CSF1R* transgene was measured using Zeiss blue 2012 edition. Average values with standard deviation graphs were plotted using GraphPad Prism 7 software. All results were analysed for statistical significance with a non-parametric Mann Whitney test. Spleens were cut into halves for flow cytometric analysis or immunofluorescence staining. All other tissues were fixed in 4% PFA for immunofluorescence staining as described above.

### 2.9 microCT scanning of bones and TRAP staining

Seventeen left femur bones were subjected to micro-CT (computed tomography) scanning to analyse bone volume and trabecular number. Femurs were fixed in 4% PFA overnight and stored in 70% ethanol at 4 °C until embedding in 0.5% agarose gel in a universal tube for scanning. Skyscan 1172 at 60 kV, 150 μA at a resolution of 5 μm was used. Restructuring 3D image was obtained using CTVox programme.

Tartrate-resistant acid phosphatase (TRAP) staining was performed as described previously (Sauter et al., 2014). Briefly, right femur bones were decalcified, embedded, and sectioned by the Royal (Dick) School of Veterinary Studies Clinical Pathology Laboratory (University of Edinburgh, UK). Four-micrometer thick sections were stained with an acid phosphatase leukocyte kit (Sigma) according to the manufacture's protocol. The staining results were observed by Nikon Ni2 bright field microscopy and images were taken under 10 × objective lens. The positively-stained osteoclasts were counted following identification using their red colour, location in resorption pits, large size, distinctive shape and presence of multiple nuclei. Two sections from each of 5–6 treated and control birds bird were stained and 3–5 views were imaged for quantification of osteoclasts. Statistical differences were analysed with a non-parametric Mann Whitney test.

## 3 Results

### 3.1 Production of two monoclonal antibodies to chicken CSF1 and prediction of CSF1 isoforms

**Fig. 1:**
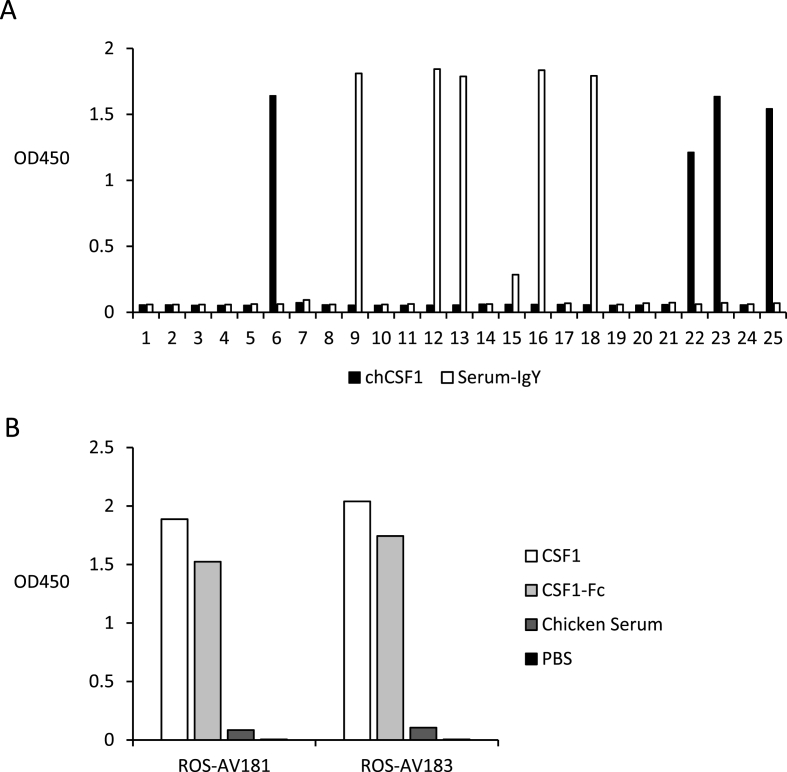
(A) Screening of hybridoma supernatant by indirect ELISA. Clone 6, 22, 23 and 25 recognised chCSF1 but not the IgY fusion partner. Clone 6 (ROS-AV181) and 22 (ROS-AV183) were successfully cloned. (B) ROS-AV181 and 183 recognised non-tagged chCSF1 and chCSF1-Fc but not the IgY-Fc fusion partner in an indirect ELISA.

To generate anti-CSF1 monoclonal antibodies, mice were immunized with chicken CSF1-Fc. Spleens were extracted and fused with myeloma cells by standard protocols. Hybridoma supernatants were screened to identify clones that produced antibodies recognising CSF1-Fc, but not the IgY fusion partner, in an indirect ELISA (Fig. 1A). Two of the positive hybridomas, designated as ROS-AV181 and ROS-AV183, were successfully cloned and tested in a range of applications. Fig. 1B shows these two mAbs recognise recombinant chCSF1 as well as CSF1-Fc but do not bind IgY-Fc as tested by indirect ELISA.

**Fig. 2:**
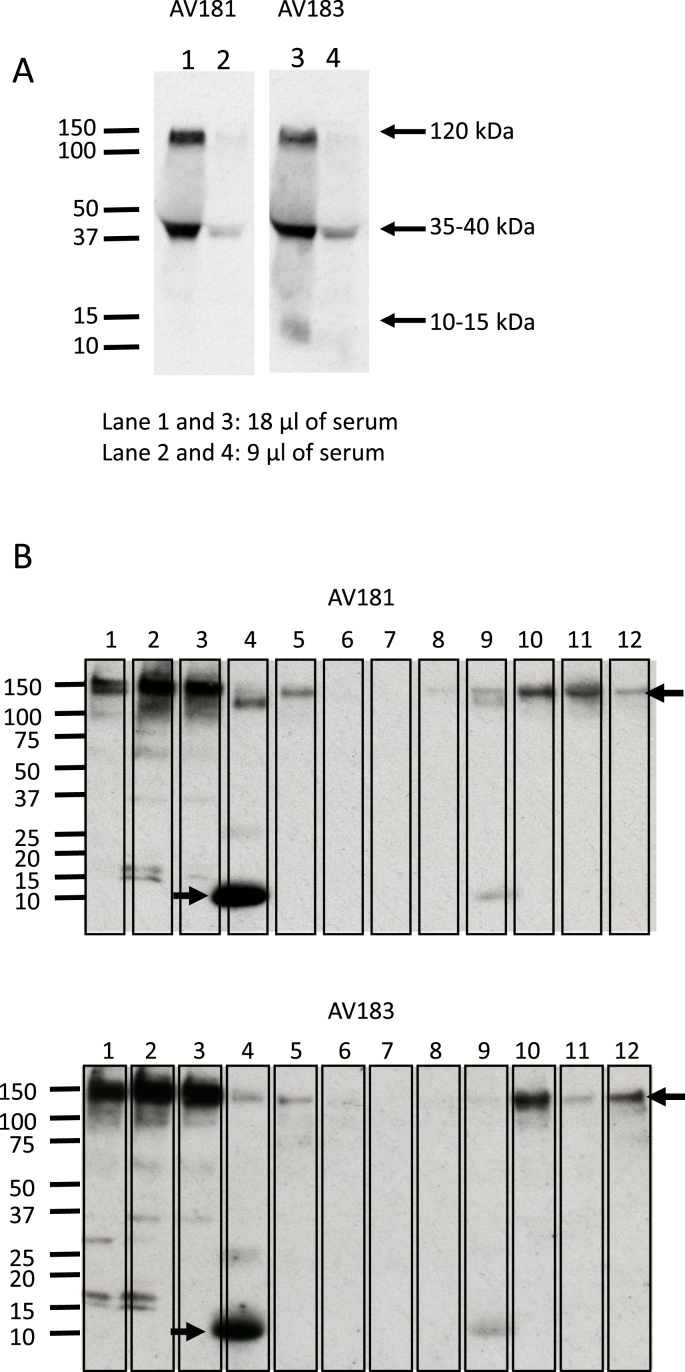
Western Blot on serum samples and tissue lysates using anti–CSF–1 (ROS-AV181 or ROS-AV183). (A) Western blot showing the two isoforms of circulating CSF1 in chicken serum. Lane 1 and 3, 18 μl of serum; lane 2 and 4, 9 μl of serum. (B) Western blot showing isoforms of CSF1 in tissue lysates. 1. Thymus, 2. Bursa, 3. Spleen, 4. Bone Marrow, 5.Cecal Tonsil, 6. Brain 7. Muscle, 8. Heart, 9. Lung, 10. Kidney, 11. Liver, 12. Jejunum.

In mammals, the CSF1 locus encodes at least three mRNA splice isoforms through differential splicing. The protein products include 3 biologically active isoforms: a membrane-spanning cell-surface glycoprotein, and secreted glycoprotein and proteoglycan isoforms. In mice, the relative importance of these isoforms has been explored through transgenic (re)expression in *Csf1*^op/op^ mice that lack endogenous CSF1 (Nandi et al., 2006; Ryan et al., 2001); (Dai et al., 2004). The genomic structure of CSF1 is similar in chickens, and initial characterization of *Csf1* mRNA indicated that all three isoforms were likely to exist in birds (Garceau et al., 2010). Western blot analysis of serum (Fig. 2A) with the two different anti-CSF1 antibodies confirmed the presence of two major bands, consistent with the secreted proteoglycan (monomeric Mr of ca 120 kDa) and glycoprotein (monomeric Mr of ca. 35–40 kDa) isoforms. A faint low Mr band around 13–15 kDa could be the core, unglycosylated and monomeric active CSF1 molecule, or may be a degradation product. In extracts of tissues (Fig. 2B), the high Mr, presumptive proteoglycan isoform was dominant and was most abundant in the three immune tissues tested, namely thymus, bursa and spleen. The low Mr band detected in the circulation was notably highly expressed in bone marrow.

### 3.2 Localization of CSF1 in the immune tissues and its expression on BMDM

**Fig. 3:**
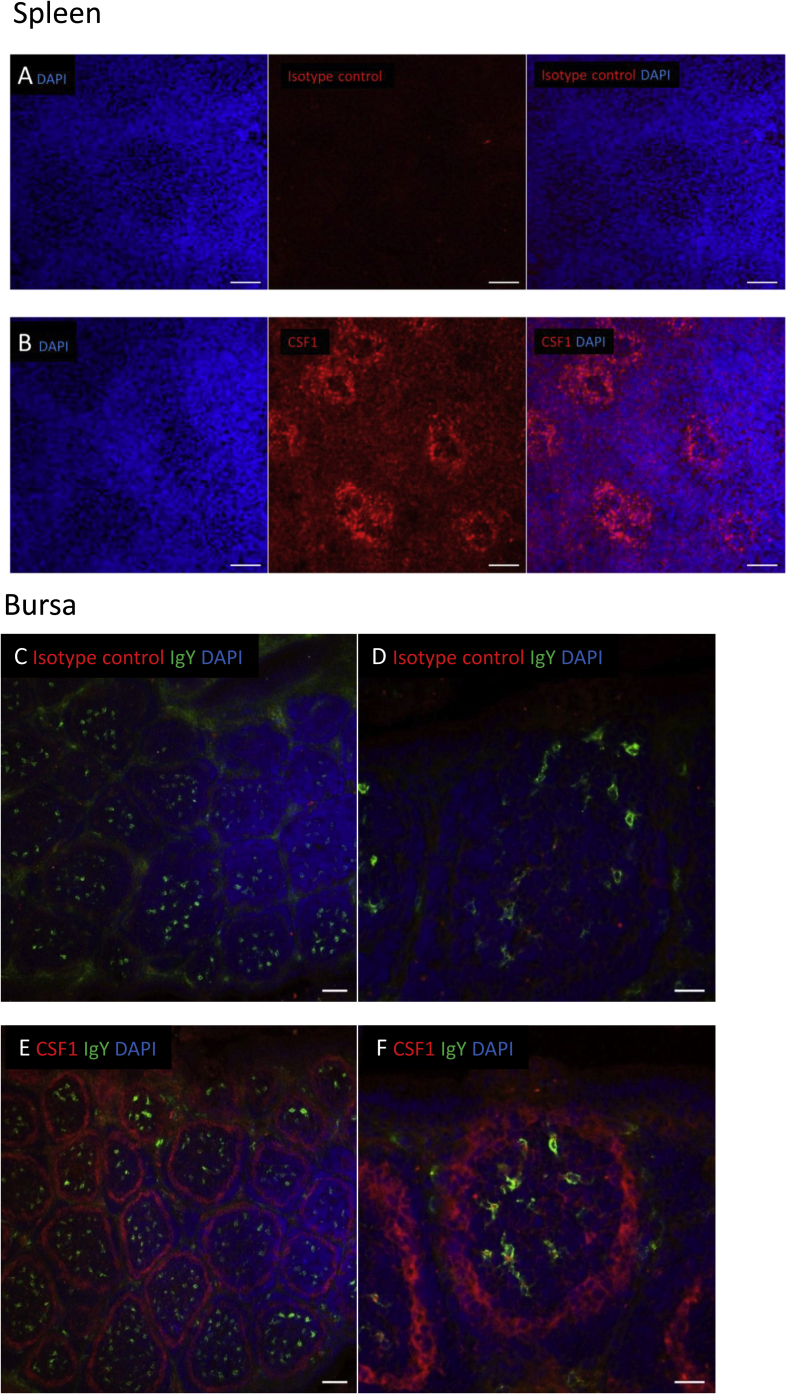
Localization of CSF1 in the immune tissues. Tissue sections were processed as in Materials and Methods. (A and B) CSF1 staining of spleen from a 16 week old chicken. CSF1 staining is localised to the ellipsoid surrounding the penicillar capillary. CSF1 (Red), and DAPI (blue), scale bar = 20 μm. (C–F) CSF1 staining on bursa of Fabricius from a 3 day old chick. CSF1 staining is localised to the cells at the cortico-medullary junction. CSF1 (Red), IgY (green) and DAPI (blue). (C and E) Objective lens 20 × ; scale bar = 20 μm. (D and F) Objective lens 40 × ; scale bar = 20 μm. (For interpretation of the references to colour in this figure legend, the reader is referred to the Web version of this article.)

To determine the location of CSF1 in the immune tissues, we carried out immunofluorescence staining of the spleen and bursa. In the spleen (Fig. 3A and B), CSF1 staining was concentrated to the splenic ellipsoid, the major location of active phagocytosis of blood-borne particles in the avian spleen, with some functional similarities to the marginal zone of rodent spleen (Jeurissen, 1993; Zhang et al., 2015). In the bursa (Fig. 3C–F), CSF1 expression was localised to the cortico-medullary boundary, which we have previously shown to contain a prominent population of TIM4^+^ macrophages (Hu et al., 2019).

**Fig. 4:**
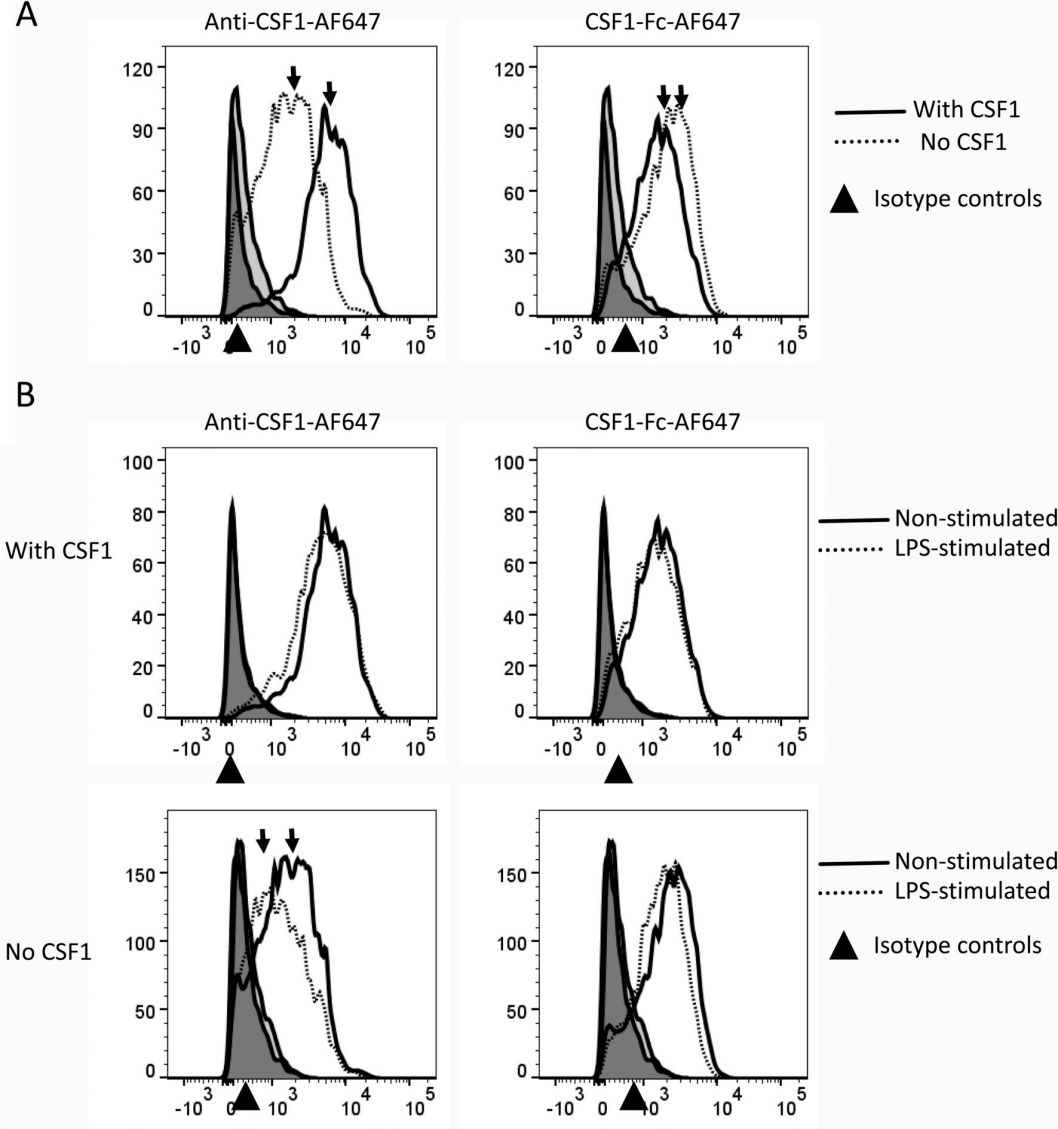
Flow cytometric analysis of surface expression of CSF1R on BMDM. BMDMs were cultured with CSF1 for 6 days. At this point, half of the cells were starved of CSF1 overnight (no CSF1) and the other half were cultured with CSF1 (with CSF1). Following a further 24 h of culture, BMDM were stimulated with LPS for 3 h. Cells were stained with either anti-CSF1-AF647 or CSF1-Fc-AF647. All histograms are representatives of three independent experiments. (A) Comparison of the staining profiles of CSF1 and CSF1-Fc between cells starved of CSF1 (dotted line) or cultured with CSF1 (solid line). (B) Comparison of the staining profiles between cells non-stimulated (solid line) or stimulated with LPS (dotted line) for 3 h. Cells were cultured with CSF1 or starved of CSF1 as in A. ▲ Isotype controls.

In separate *in vitro* studies, we generated RNAseq profiles from chicken macrophages grown in CSF1 (Freem et al., 2019). The data indicated that, unlike mouse bone marrow-derived macrophages, but in common with all other mammalian species studied including rats and humans, chicken macrophages grown in CSF1 express high levels of their own growth factor. We used the RNAseq data to investigate the nature of the *CSF1R* transcript(s) in chicken BMDM, confirming that they make the longer form of the mRNA encoding an integral membrane form of the protein (Freem et al., 2019). Based upon the mRNA expression, we examined whether the anti-CSF1 antibody would bind to the surface of chicken BMDM. To remove exogenous CSF1, cells were washed and starved overnight of CSF1. In contrast to mouse BMDM, which require exogenous CSF1 for survival (Griggs and Zinkewich-Peotti, 2009; Sester et al., 1999), chicken BMDM remain viable after CSF1 removal. To detect the cell surface expression of CSF1R, recombinant CSF1-Fc labelled with Alexa Fluor® 647 (CSF1-Fc-AF647) was used. The use of the ligand to analyse surface CSF1R has been applied previously in mice and rats (Hawley et al., 2018; Pridans et al., 2018; Rojo et al., 2019). Fig. 4 shows that the entire BMDM population stained for surface CSF1 (as detected by anti-CSF1 antibody) or CSF1R (as detected by CSF1-Fc-AF647). Panel A shows that the binding of the labelled ligand was only marginally increased when the cells were starved of exogenous CSF1 overnight, in contrast to the massive upregulation seen in CSF1-starved mouse BMDM (Sester et al., 1999). On the other hand, cell surface CSF1 immunoreactivity was greatly reduced in the cells incubated without CSF1 overnight. In mouse macrophages, stimulation with bacterial lipopolysaccharide (LPS), or other toll-like receptor agonists, causes rapid release of CSF1R from the cell surface (Sester et al., 1999). Like mouse, chicken BMDM respond to LPS with a cascade of inflammatory gene activation (Garcia-Morales et al., 2015), but there was no impact after 3 h treatment on the level of surface CSF1-Fc-AF647 binding (Fig. 4B). Instead, LPS caused a significant reduction in cell surface CSF1 in the CSF1-starved cells, but not in those that have been maintained in CSF1 (Fig. 4B).

### 3.3 Comparative analysis of circulating CSF1 levels in layers and broilers as detected by capture ELISA

**Fig. 5:**
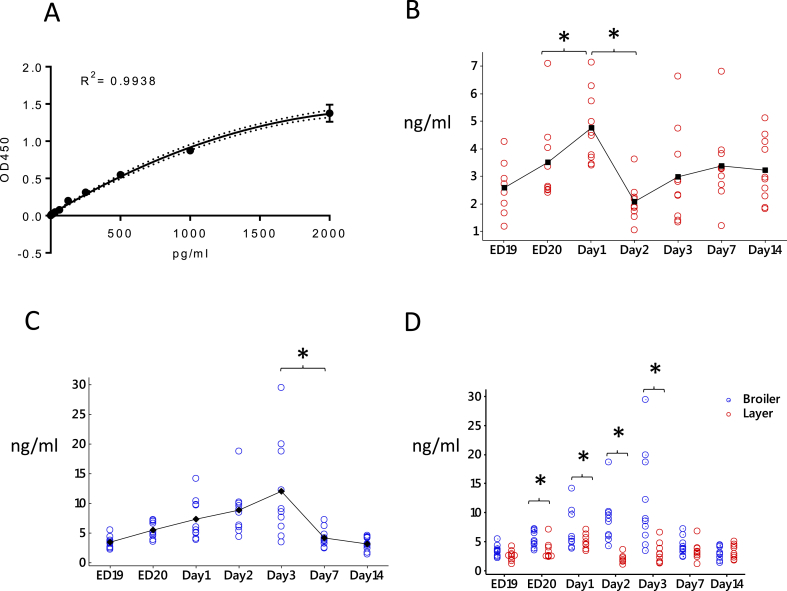
Development of a capture ELISA assay and comparison of CSF1 levels in sera between broiler and layer birds. (A) Standard curve of capture ELISA assay. (B and C) CSF1 concentrations in sera from broilers (panel B) and layers (panel C) from embryonic day (ED) 19 to day 14 after hatch (n = 10). (D) Comparison of CSF1 concentrations in sera from broilers (Blue) and layers (Red). A non-parametric Mann–Whitney test was used to analyse differences of CSF1 levels between time intervals (panels B and C) and bird types (panel D). *P* values < 0.05 were considered significant. (For interpretation of the references to colour in this figure legend, the reader is referred to the Web version of this article.)

We next tested combinations of the available monoclonal antibodies to generate a sandwich capture ELISA, using recombinant bacterial-expressed chicken CSF1 as a standard. The optimized assay had a detection limit around 62.5 pg/ml to 2 ng/ml (Fig. 5A). In mice, circulating CSF1 was reported to be highest in fetal and neonatal serum, around 30–40 ng/ml, and declined in the postnatal period (Roth and Stanley, 1996). In mice, both CSF1 and CSF1R mutations produce severe post-natal growth retardation (Chitu and Stanley, 2006). Broiler chickens have been selected for rapid post-hatch weight gain. We therefore used the CSF1 ELISA to examine expression during embryonic development and the phase of rapid post-hatch growth and to compare the time courses of circulating CSF1 in layer and broiler birds (Fig. 5 B- D). The circulating CSF1 concentration prior to hatch was 10-fold lower than reported in mice, around 3–5 ng/ml in late incubation. The level increased transiently in both layers and broilers around the day of hatch. On average the level of CSF1 in broilers was higher than in layers, but more variable. The differences between broiler and layer birds were significantly greater from ED20 to post hatch D3.

### 3.4 Confirmation of neutralising activity of the anti-CSF1 (ROS-AV183) antibody *in vitro*

**Fig. 6:**
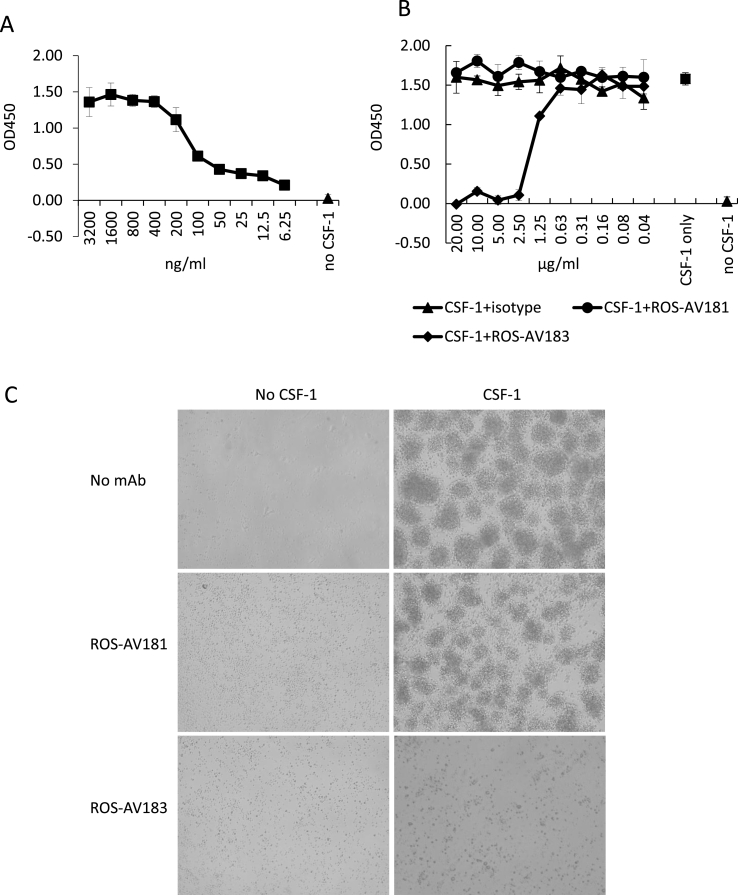
Neutralising effect of mAbs to CSF1. (A) Chicken CSF1 was titrated out on Ba/F3 cells stably transfected with CSF1R (Ba/F3-CSFR) to determine a suitable concentration for neutralising assay. 400 ng/ml was chosen. (B) Ba/F3-CSFR were cultured with CSF1, CSF1 pre-incubated with either ROS-AV181 or 183, mouse IgG1 control or without CSF1 for 72 h before performing MTT assays. Cells cultured with CSF1 or CSF1 pre-incubated with ROS-AV181 or isotype control proliferated. Cells cultured with CSF1 pre-incubated with ROS-AV183 were dead when >2.5 μg/ml of antibody was used. Assays were carried out in triplicate and representative data from three independent experiments are shown. (C) Chicken bone marrow cells were cultured with CSF1 pre-incubated with mAbs, CSF1 only or without CSF1. Original magnification objective lens 10X.

To assess whether antibodies would block CSF1 action, we used Ba/F3 growth factor-dependent cells expressing the chicken CSF1R (Garceau et al., 2010). Fig. 6A shows the dose-response curve for chicken CSF1 using this assay. Note that the minimal active concentrations (around 5 ng/ml) are in the range of serum concentrations measured above. Fig. 6B shows that one of the anti-CSF1 antibodies we generated, ROS-AV183, was able to completely block the action of a just-maximal concentration of chicken CSF1. We confirmed the biological activity by demonstrating that ROS-AV183, but not ROS-AV181, blocked the ability of CSF1 to generate macrophages from bone marrow progenitors (Fig. 6C). The relatively stoichiometry (2.5 μg/ml blocking 400 ng/ml of CSF1) suggested that the antibody could have sufficient affinity to block actions of CSF1 *in vivo.*

### 3.5 Anti–CSF–1 antibody treatment in hatchlings

**Fig. 7:**
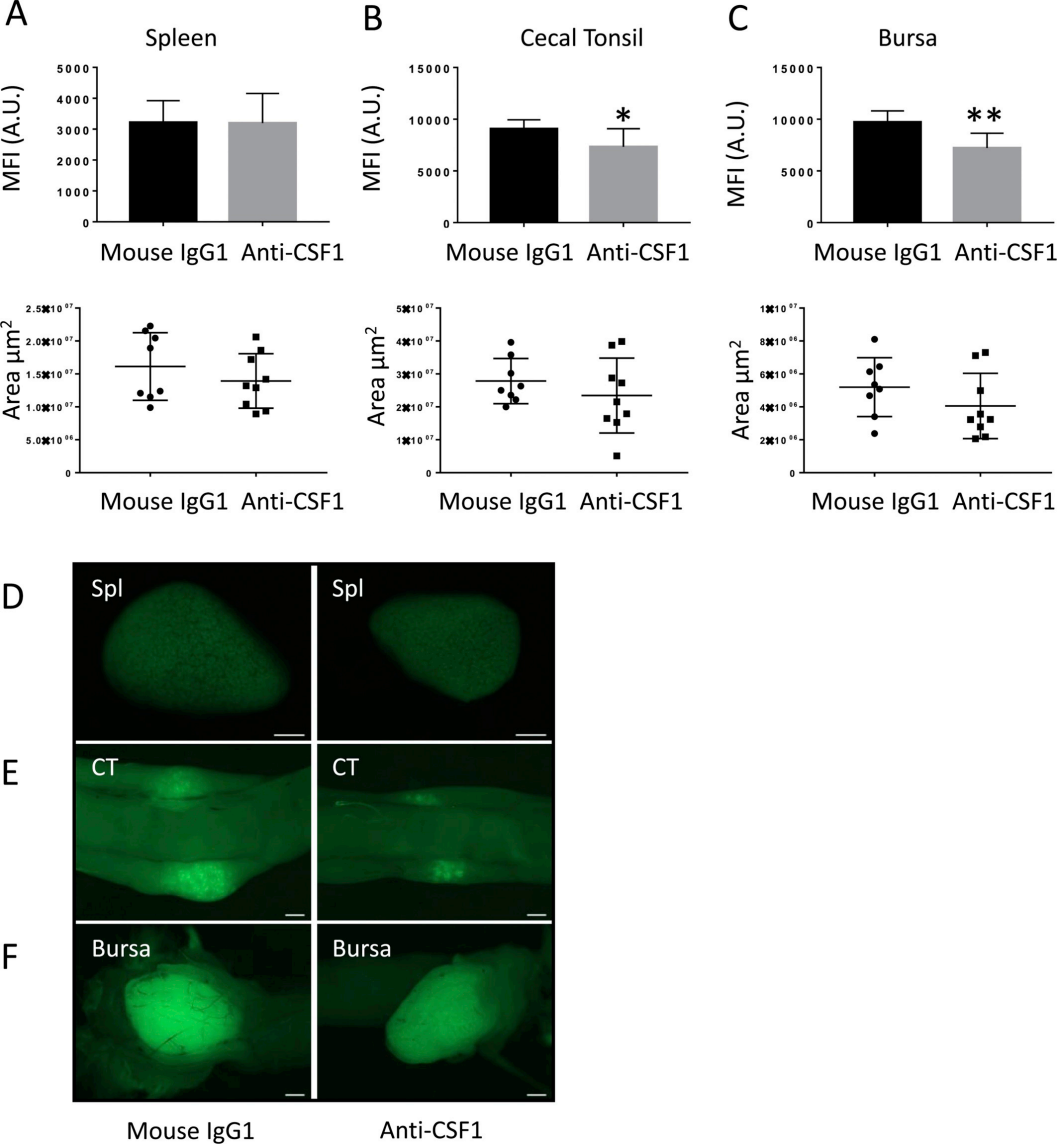
The effect of anti-CSF1 treatment on lymphoid organs in hatchling birds. *CSF1R-*eGFP reporter (MacGreen) birds received anti-CSF1 (ROS-AV183) or mouse IgG1 control antibody subcutaneously for four consecutive days from day of hatch and were sacrificed on day 7. (A–C) Mean fluorescence intensity (MFI) and surface area between the two groups obtained using Zen blue 2012 software, showing mean values of spleen, cecal tonsil and bursa. Error bars represent standard error of the mean. Significance was indicated by *P < 0.05 using a Mann–Whitney test. (D–F) Representative whole mount images of lymphoid tissues spleen, cecal tonsil and bursa obtained using Zeiss Axio Cam HR microscope, scale bar = 1 mm. (For interpretation of the references to colour in this figure legend, the reader is referred to the Web version of this article.)

To test the impact of inhibition of CSF1 *in vivo*, we utilised a *CSF1R* reporter line described previously (Balic et al., 2014) and treated newly-hatched birds with the neutralising antibody ROS-AV183 for four consecutive days. Treated birds showed no signs of illness and developed normally. By contrast to the impact of anti-CSF1 treatment in mice (Wei et al., 2005), there was no effect on post-hatch body weight gain (data not shown). To examine the transgene expression in major lymphoid tissues including cecal tonsil, bursa and spleen, the mean fluorescence intensity (MFI) and surface area was obtained from whole mount images analysis using Zeiss blue software. The surface area of different organs was correlated to body weight of each bird, regardless of treatment. There was no significant difference in the MFI or the surface area of spleen (Fig. 7A) but a significant decrease in the MFI of transgene expression was detected in both cecal tonsil (Fig. 7B) and bursa (Fig. 7C) after anti-CSF1 treatment. Surface area of bursa and cecal tonsil tissue remained unaffected. Representative images comparing treated and untreated spleen, cecal tonsil and bursa are shown in Fig. 7D–F. The decrease in *CSF1R*-transgene positive cells in cecal tonsil and the bursa of Fabricius from birds treated with anti-CSF1 was further detected by confocal microscopy using anti-eGFP antibody (Supplemental Material Fig. S1).

**Fig. 8:**
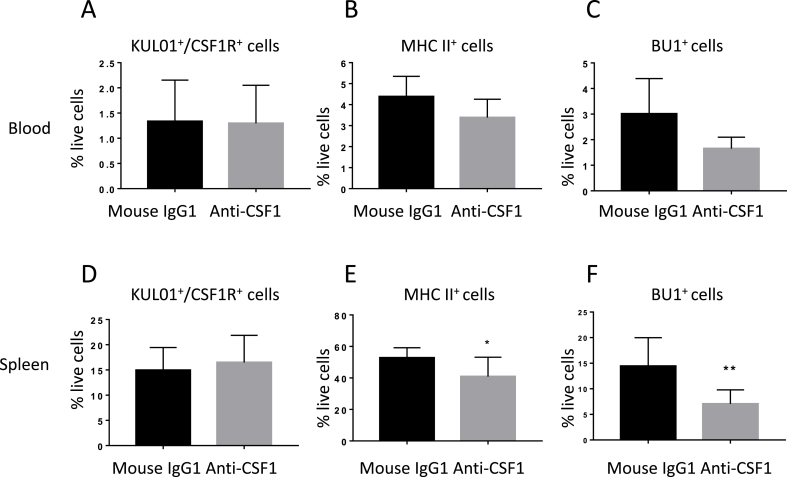
The effect of anti-CSF1 treatment on blood leukocytes and splenocytes. Blood leukocytes and splenocytes were isolated, stained with KUL01, anti-MHC class II and anti-Bu-1 and analysed by flow cytometry. (A–C) The profiles of peripheral blood cells from *CSF1R*-eGFP birds treated with anti-CSF1 (n = 8) or with mouse IgG1 isotype control antibody (n = 6). Significance is indicated by *P < 0.05 using a Mann–Whitney test. (D–F) The profiles of spleen cell suspension from *CSF1R*-eGFP birds treated with anti-CSF1 (n = 9) or an isotype control antibody (n = 8). Significance is indicated by ***P < 0.001 using a Mann–Whitney test.

Fig. 8 shows the profiles of peripheral blood mononuclear cells from *CSF1R*-eGFP birds treated with anti-CSF1 or an isotype control antibody. The blood cells were stained for the monocyte marker KUL01, B cell marker BU.1 and class II MHC. The proportions of the different cell populations were variable between birds, regardless of treatment. There was no significant effect of anti-CSF1 on CSF1R^+^ KUL01^+^ monocytes (Fig. 8A). Both MHC class II (Fig. 8B, which labels both B cells and monocytes) and Bu-1 (Fig. 8C) staining indicated a marginal reduction in circulating B cells. In the spleen, anti-CSF1 treatment had no impact on the CSF1R^+^, KUL01^+^ populations (Fig. 8D). However, the numbers of MHC II^+^ cells and Bu-1^+^ cells in the spleen were significantly reduced (Fig. 8E and F).

**Fig. 9:**
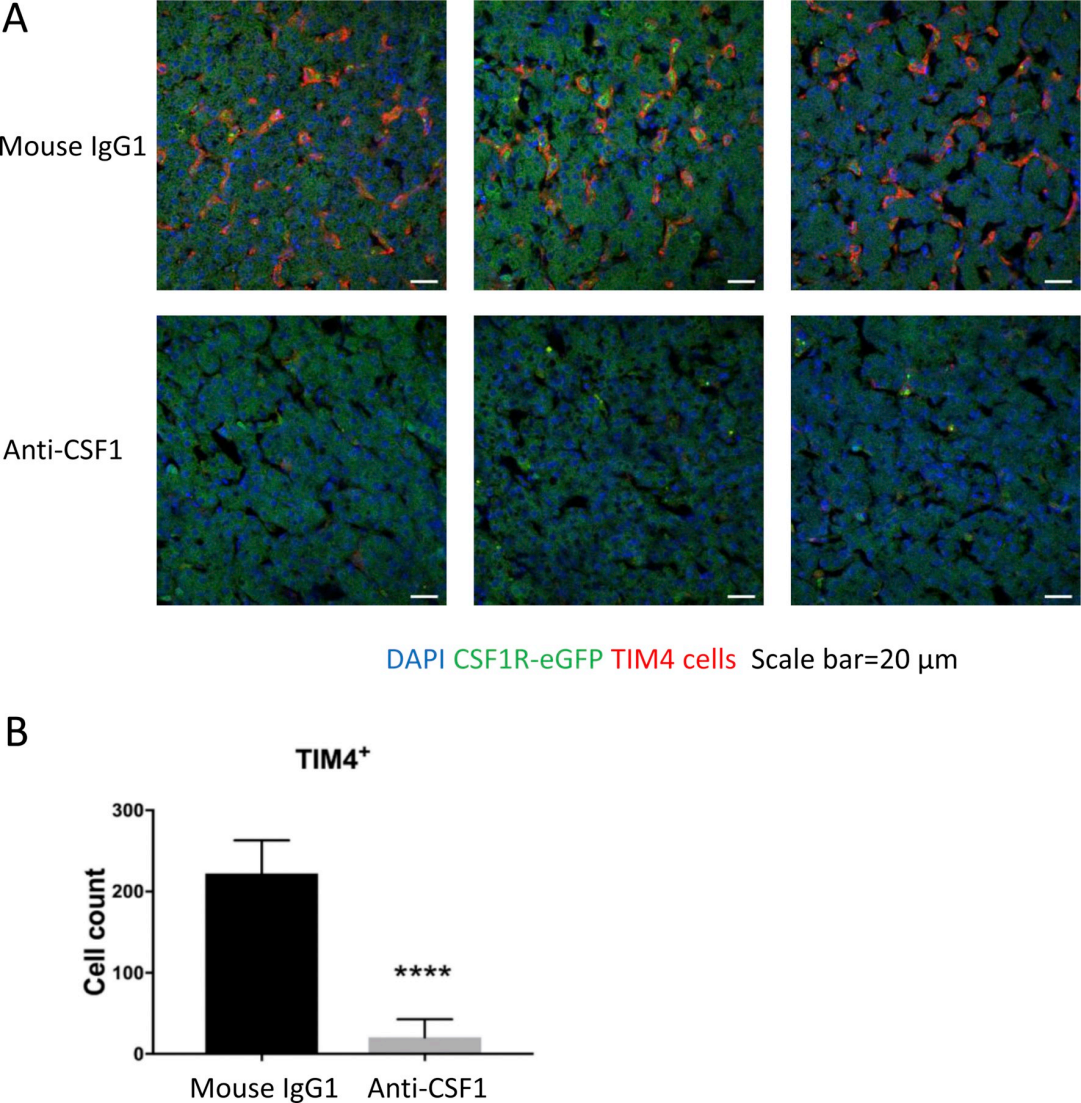
The effect of anti-CSF1 treatment on TIM4^+^ Kupffer cells. (A) Confocal images of Kupffer cells as detected by anti-chicken TIM4 mAb (red) staining in liver tissue *(n = 8/9)* using LSM 710 inverted microscope. Representative image from three birds from each treatment. Green indicates *CSF1R* transgene expression, red indicates TIM4^+^ cells and blue denotes nuclei. Scale bar = 20 μm. (B) The numbers of TIM4^+^ Kupffer cells per image were quantified using ImageJ from 5 different images from 5 different regions of liver sample per bird per treatment at 40 × magnification. The graph indicates the average count of TIM4^+^ cells (red channel) per image with standard deviation between the mouse IgG1 and anti-CSF1 treated group. **** indicates significance at *P* < 0.0001 using a Mann–Whitney test. (For interpretation of the references to colour in this figure legend, the reader is referred to the Web version of this article.)

The liver tissue resident macrophage population (Kupffer cells) in mice is rapidly depleted by treatment with anti-CSF1R antibody (MacDonald et al., 2010) or anti-CSF1 (Lin et al., 2019). In the chick, the *CSF1R*-reporter is not expressed by Kupffer cells (KC), although they express high levels of *CSF1R* mRNA (Hu et al., 2019). To assess the effect of anti-CSF1 in the liver, we used an antibody against the KC marker TIM4 (Hu et al., 2019). Fig. 9 shows that TIM4^+^ cell population was almost completely ablated by anti-CSF1 treatment. Representative immunohistochemistry images from three individual animals are shown in Fig. 9A. Fig. 9B shows that the average TIM4^+^ cell number per image was significantly reduced in the anti-CSF1 treated group compared to the mouse IgG1 control group.

**Fig. 10:**
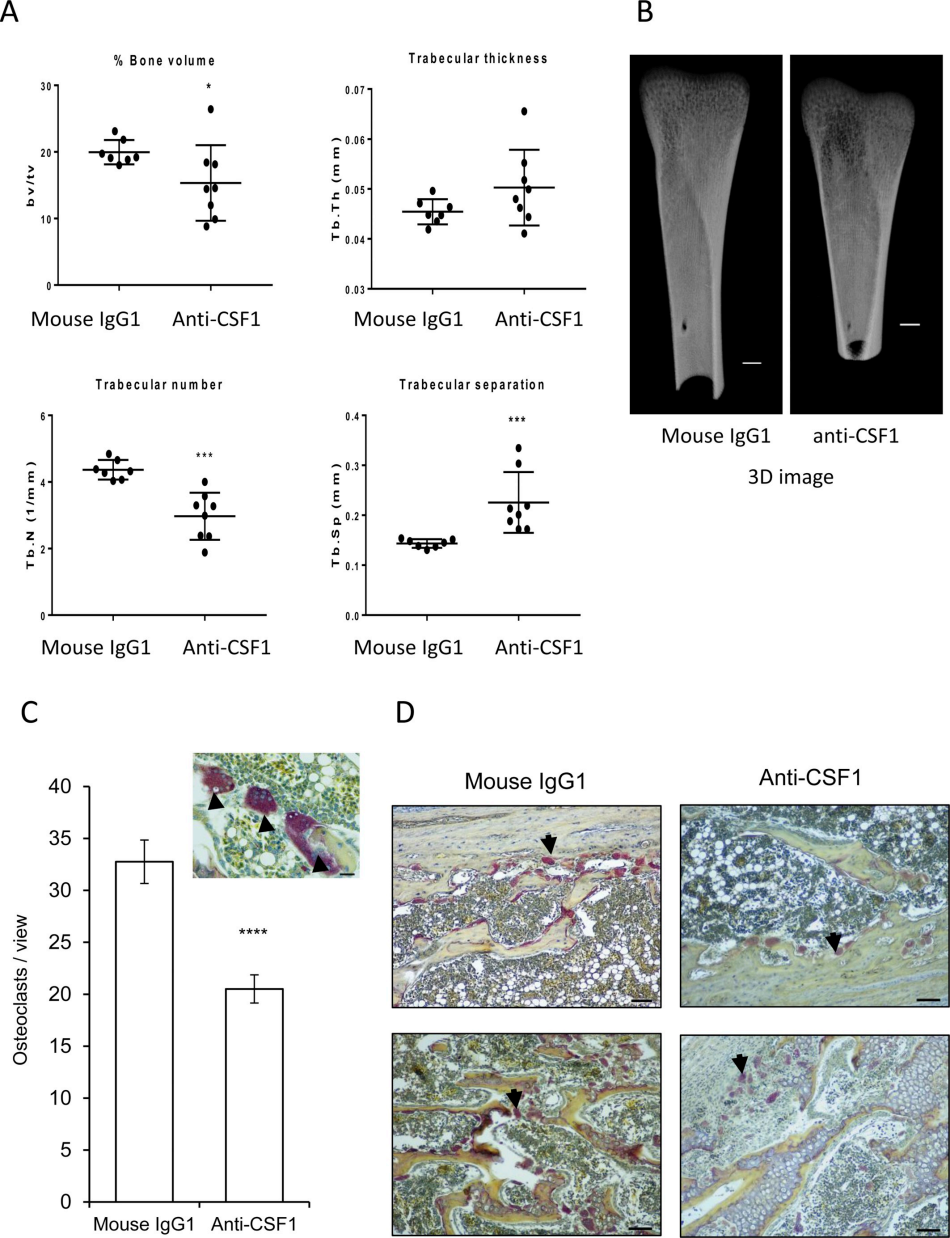
The effect of anti-CSF1 treatment on bone. (A) Comparison of bone volume and trabecular thickness, number and separation by microCT scanning of femur bones between chicks treated with mouse IgG1 or anti-CSF1 (N = 7/8). Significance is indicated by *P < 0.05, using a Mann–Whitney test. (B) Representative 3D images from birds treated with mouse IgG1 and anti-CSF1 antibody obtained using CTVox programme. Scale bar = 1.0 mm. (C) Comparison of TRAP^+^ osteoclasts (black arrows) in the bone between mouse IgG1 and anti-CSF1 treated groups. Significance is indicated by ***P < 0.005, using a Mann–Whitney test. (D) Representative TRAP staining images of mouse IgG1 and anti-CSF1 treated birds. Scale bar = 50 μm.

As CSF1 mRNA and protein were highly-expressed in bone marrow (Fig. 2B), and treatment of hatchling birds with CSF1-Fc increased bone density, trabecular thickness and trabecular number within one week (Garceau et al., 2015), we examined the impact of anti-CSF1 treatment on the same parameters. The bone volume and trabecular number were significantly reduced in the treated birds, and correspondingly, the trabecular separation was increased (Fig. 10A and B). TRAP staining results showed that bone osteoclasts (OCL) were significantly reduced by anti-CSF1 treatment (Fig. 10C and D).

## 4 Discussion

We have generated anti-chicken CSF1 antibodies and utilised them to study the biology of CSF1 in the chicken. In mammalian systems, there is surprisingly little information on the location of CSF1 in tissues, and to our knowledge, no published staining with an anti-CSF1 monoclonal in any mammalian species. Somewhat at odds with the protein expression we have observed by Western blotting, *CSF1* mRNA is widely expressed in cells and tissues in a recently generated chicken expression atlas (www.biogps.org/chickenatlas) (Bush et al., 2018). Nevertheless, chicken BMDM had the highest level of *CSF1* mRNA expression of any cell or tissue assay including fibroblasts (Garceau et al., 2010). The difference between the mRNA and protein data likely reflects differences in production of secreted versus cell membrane forms of the protein in different tissues and cell types. A *Csf1*-*lacZ* reporter gene was used previously in mice to locate CSF1-expressing cells (Ryan et al., 2001). They reported the presence of *Csf1*-*lacZ* positive cells in the marginal zone of spleen and to a lesser extent in red pulp, and in outer cortical areas of lymph nodes, where macrophages are concentrated. A recent study located CSF1 in mouse lymph nodes to lymphatic endothelial cells and confirmed the function in maintenance of the subcapsular sinus macrophage population (Mondor et al., 2019). In most other tissues, the LacZ reporter was associated with epithelial and glandular structures. These authors concluded that CSF1 was mainly expressed by cells of mesenchymal origin. Indeed, the source of CSF1 in mice *in vivo* is commonly attributed to mesenchymal (endothelial cells, fibroblasts, osteoblasts, adipocytes) and epithelial cells (Stanley and Chitu, 2014). In mice, *Csf1* mRNA is not expressed by BMDM grown in CSF1, nor by any isolated tissue macrophage population assayed by the ImmGen consortium (www.immgen.org). However, *CSF1* mRNA was shown to be inducible during differentiation in human monocytes by growth factors and phorbol esters (Horiguchi et al., 1986, 1987, 1988). The highest level of expression of *CSF1* mRNA in the FANTOM5 human transcriptional atlas is in monocyte-derived macrophages (Consortium et al., 2014). Csf1 mRNA is also expressed at high levels in pig (Freeman et al., 2012) and rat (Rojo et al., 2019) BMDM. Using the anti-CSF1 antibody, we confirmed here that chicken BMDM also express CSF1 on their surface, and the level appears to be regulated further by exogenous CSF1. Unlike mouse BMDM, once progenitors have differentiated in response to CSF1 the resulting chicken BMDM do not depend on exogenous CSF1 for survival, consistent with a role for autocrine CSF1.

Three species of CSF1 were detected in serum by Western blotting (Fig. 2A). We used the anti-CSF1 antibodies to develop an ELISA and to measure the concentration of circulating CSF1. The level we detected, in the low ng/ml range, is consistent with the apparent saturation of the CSF1R receptor with ligand in bioassays (Fig. 6B). We previously demonstrated that the availability of CSF1 in hatchling birds is not saturating for monocyte-macrophage generation. The administration of exogenous CSF1-Fc produced a massive expansion of circulating and tissue cell populations expressing *CSF1R* reporter genes (Garceau et al., 2015). Here we characterized a neutralising antibody and showed that it was able to block CSF1 biological activity *in vitro* (Fig. 6). We were therefore able to demonstrate that CSF1 was essential for the development and/or maintenance of tissue macrophages.

We also analysed the impact of anti-CSF1 on the development of the bone. Anti-CSF1 treatment produced a reduction in overall bone density and trabecular volume (Fig. 10), the reciprocal of the impact of CSF1-Fc treatment (Garceau et al., 2015). In mice, CSF1R is expressed by bone-resorbing osteoclasts (OCL). CSF1-deficient mice are osteopetrotic, and OCL are ablated in adult mice treated with anti-CSF1R leading to an increase in bone density and trabecular volume. Paradoxically, treatment of mice with CSF1-Fc also increased bone density despite an increase in OCL number (Gow et al., 2014). This apparent conflict probably arises because ossification mediated by osteoblasts depends upon a novel macrophage populations that is also CSF1-dependent (Batoon et al., 2019). Our studies in chicken are conducted in juvenile birds with rapid bone growth. We speculate that the elevated CSF1 at hatch (Fig. 3), and the higher levels apparent in the rapidly growing broiler birds, may have a regulatory function in skeletal growth.

In mice, although exogenous CSF1 can massively expand blood monocyte numbers (Yao et al., 2014), CSF1 is not required for monocyte production. Anti-CSF1R antibody administration to adult mice did not alter the number of monocytes, although it did selectively block their differentiation to a non-classical phenotype (Gow et al., 2014). There was no effect of anti-CSF1 on monocyte numbers in the treated chick (Fig. 8). At present, we do not have any evidence for functional heterogeneity of chicken monocytes, and anti-CSF1 did not alter expression of the markers we currently have available (MHC Class II, KUL01).

Consistent with the impact of anti-CSF1R in mice, anti-CSF1 treatment did impact on some tissue macrophage populations. There was almost complete loss of the TIM4^+^ KC population in the liver (Fig. 9), and partial loss of the *CSF1R*-transgene positive cells in the bursa and cecal tonsil (Fig. 7). Given the very high level of CSF1 protein in lymphoid organs, the antibody may be less able to compete with endogenous ligand in these tissues. In the peripheral blood and in spleen and bursa, there was an apparent selective effect on B cell numbers. A selective defect in B cell lymphopoiesis was also reported in analysis of a *Csf1* knockout mouse (Harris et al., 2012). Transgenic expression of the proteoglycan form of CSF1 was able to restore B cell numbers in BM, blood and spleen (Nandi et al., 2006). Clearly the biology is different in birds, in which B cells are produced by the bursa and no classical lymph node structures have been identified to date. There is no evidence that *CSF1R* is expressed in chicken B cells (Balic et al., 2014; Hu et al., 2019). Accordingly, we suggest that CSF1-dependent macrophages in the bursa and spleen provide trophic factors that can influence the development or survival of B cells. One macrophage-dependent candidate regulator of bursal B cell development is B cell activating factor (BAFF, TNFSF13B) (Koskela et al., 2004). In the chicken expression atlas (www.biogps.org/chickenatlas), *Tnfsf13b* mRNA is enriched in bursa, expressed by BMDM, and strongly induced following stimulation with LPS.

## 5 Conclusion

We have produced two mouse monoclonal antibodies to chicken CSF1 and developed one capture ELISA assay to quantify native CSF1. Antibody ROS-AV183 neutralised the bioactivities of chCSF1 *in vitro*. Treatment of hatchlings using this neutralising antibody *in vivo* impacted on some tissue macrophage populations. These antibodies are new tools to study the roles of CSF1 in birds.
