# Supplementary material for: Regulation and function of macrophage colony-stimulating factor (CSF1) in the chicken immune system
Source: Dev Comp Immunol. 2020 Apr;105:103586. doi: 10.1016/j.dci.2019.103586 (PMC6996135; doi:10.1016/j.dci.2019.103586)
Supplement: Multimedia component 2 [file mmc2.pptx]

## Slide 1
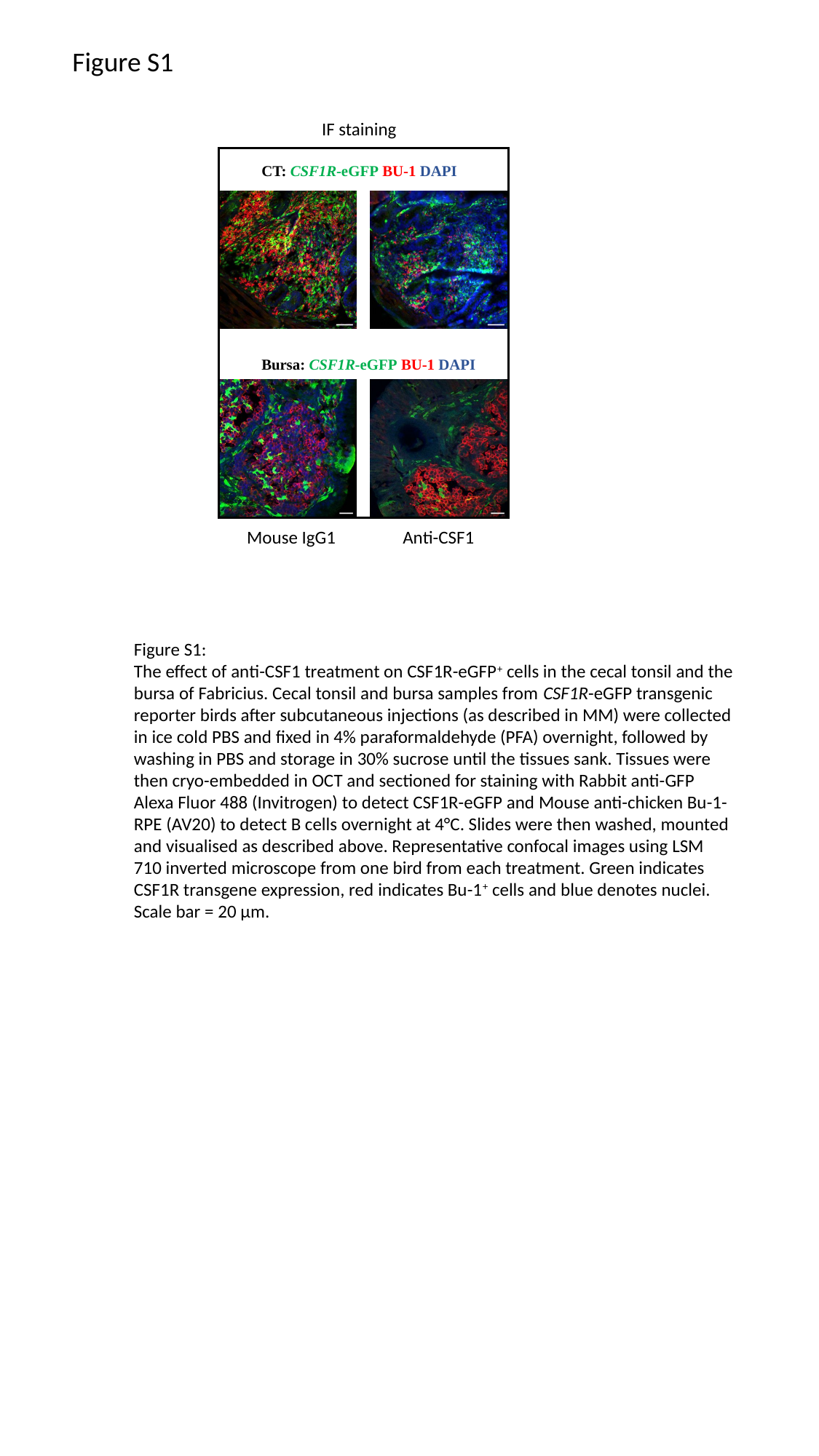

Figure S1
IF staining
CT: CSF1R-eGFP BU-1 DAPI
Bursa: CSF1R-eGFP BU-1 DAPI
Mouse IgG1
Anti-CSF1
Figure S1:
The effect of anti-CSF1 treatment on CSF1R-eGFP+ cells in the cecal tonsil and the bursa of Fabricius. Cecal tonsil and bursa samples from CSF1R-eGFP transgenic reporter birds after subcutaneous injections (as described in MM) were collected in ice cold PBS and fixed in 4% paraformaldehyde (PFA) overnight, followed by washing in PBS and storage in 30% sucrose until the tissues sank. Tissues were then cryo-embedded in OCT and sectioned for staining with Rabbit anti-GFP Alexa Fluor 488 (Invitrogen) to detect CSF1R-eGFP and Mouse anti-chicken Bu-1-RPE (AV20) to detect B cells overnight at 4°C. Slides were then washed, mounted and visualised as described above. Representative confocal images using LSM 710 inverted microscope from one bird from each treatment. Green indicates CSF1R transgene expression, red indicates Bu-1+ cells and blue denotes nuclei. Scale bar = 20 µm.
